# Nitric Oxide/Cyclic GMP-Dependent Calcium Signalling Mediates IL-6- and TNF-α-Induced Expression of Glial Fibrillary Acid Protein

**DOI:** 10.1007/s12031-020-01708-3

**Published:** 2020-09-22

**Authors:** Claudia Sticozzi, Giuseppe Belmonte, Maria Frosini, Federica Pessina

**Affiliations:** 1 Department of Life Sciences, University of Siena, Via Aldo Moro 2, 53100 Siena, Italy; 2 U.O.C. Anatomia Patologica, Policlinico Santa Maria alle Scotte, Viale M. Bracci, 16, 53100 Siena, Italy; 3 Department of Molecular and Developmental Medicine, University of Siena, Via Aldo Moro 2, 53100 Siena, Italy

**Keywords:** Pro-inflammatory cytokines, GFAP, NO/cGMP/Ca^2+^ signalling, Astrogliosis, Neuroinflammation, Neurological disorders

## Abstract

Astrocyte activation is characterized by hypertrophy with increased glial fibrillary acidic protein (GFAP), whose expression may involve pro-inflammatory cytokines. In this study, the effects of pro-inflammatory IL-6 and TNF-α and anti-inflammatory cytokines IL-4 and IL-10 on nitric oxide (NO)/cyclic guanosine monophosphate (cGMP) signalling, intracellular calcium concentration ([Ca^2+^]_i_) and GFAP expression were investigated. In human glioblastoma astrocytoma U-373 MG cells, IL-6 and TNF-α, but not IL-4 or IL-10, increased iNOS, cGMP, [Ca^2+^]_i_ and GFAP expression. The inhibitors of iNOS (1400 W), soluble guanylyl cyclase (ODQ) and IP3 receptors (ryanodine and 2-APB) reversed the increase in cGMP or [Ca^2+^]_i_, respectively, and prevented GFAP expression. In rat striatal slices, IL-6 and TNF-α, at variance with IL-4 and IL-10, promoted a concentration-dependent increase in Ca^2+^ efflux, an effect prevented by 1400 W, ODQ and RY/2APB. These data were confirmed by in vivo studies, where IL-6, TNF-α or the NO donor DETA/NO injected in the striatum of anaesthetised rats increased cGMP levels and increased GFAP expression. The present findings point to NO/cGMP-dependent calcium signalling as part of the mechanism mediating IL-6- and TNF-α-induced GFAP expression. As this process plays a fundamental role in driving neurotoxicity, targeting NO/cGMP-dependent calcium signalling may constitute a new approach for therapeutic interventions in neurological disorders.

## Introduction

Neuroinflammation, an inflammatory response within the brain or spinal cord, involves the participation of resident immune-like glial cells such as microglia and astrocytes, endothelial cells and different peripheral immune cells including monocytes/macrophages, leukocytes and lymphocytes (DiSabato et al. 2016). The central and peripheral immune systems are integrated and form an interdependent, bidirectionally communicating, neuro-immune network (Sredni-Kenigsbuch 2002; Pavlov et al. 2018). Indeed, the inflammatory response marking the beginning of wound healing and scarring events includes the activation of endogenous glial cells that produce pro-inflammatory mediators such as cytokines, chemokines and lipid mediators that drive neutrophil recruitment to injured tissue (Stirling et al. 2009). Subsequently, monocytes are mobilized and recruited, followed by lymphocytes. The latter, once differentiated into the Th1 category, release pro-inflammatory cytokines such as TNF-α and IL-6, which in turn, by an autocrine and paracrine process, further activate glial cells, perpetuating a vicious cycle which contributes to progression of inflammation and possible cytotoxicity (Paul and Seder 1994; Greenhalgh et al. 2020). In contrast, lymphocytes of the Th2 category release anti-inflammatory cytokines including IL-4 and IL-10, which play a significant role in amelioration of inflammation and neuroprotection (Sredni-Kenigsbuch 2002). With the use of different central nervous system (CNS) injury models, several cells and cytokines of the immune system have been convincingly implicated in the generation or modulation of reactive astrogliosis (Dantzer 2018; Escartin et al. 2019). In astrocytes and brain striatal tissue, we previously established that nitric oxide/cyclic guanosine monophosphate (NO/cGMP)-dependent Ca^2+^ mobilization from intracellular pools is part of a signalling pathway subserving the pro-inflammatory/pyrogenic effect of IL-1β (Meini et al. 2000; Palmi and Meini 2002) and that the same cascade, via activation of calmodulin and extracellular signal-regulated protein kinases (ERK1/2), is responsible for IL-1β upregulation of astrocyte proliferation (Meini et al. 2006, 2008). In addition, changes in intracellular NO and calcium levels occur in neuroinflammatory processes, leading to a number of neurodegenerative disorders including Parkinson’s and Huntington’s diseases, ischemic stroke, and amyotrophic lateral sclerosis (de Waal Malefyt et al. 1991; Linares et al. 2006; Mattson 2007). In the present study, using in vitro, ex vivo and in vivo models, we investigated the effects of TNF-α and IL-6 on the expression of glial fibrillary acidic protein (GFAP), a hallmark of astrocyte activation, and the role of the NO/cGMP/calcium signalling pathway in mediating this response.

## Materials and Methods

### Chemicals

Chemicals were purchased as follows: human recombinant IL-4, IL-10, IL-6 and rat recombinant TNF-α (ImmunoTools, Friesoythe, Germany); ^45^Ca^2+^ (DuPont NEN, Cologno Monzese, Milan, Italy); 1H-[1,2,4] oxadiazole [4,3-a] quinoxalin-1-one (ODQ), ryanodine (RY), 2-aminoethoxydiphenylborane (2-APB) and N-[(3-(aminomethyl)-phenyl]methyl]-ethanimidamide dihydrochloride (1400 W) Tocris Cookson, Bristol, UK); monoclonal mouse anti-GFAP primary antibody (Acris, Herford, Germany); fluorescein isothiocyanate (FITC)-conjugated anti-mouse secondary antibodies (Millipore, Billerica, MA, USA). All other chemicals were purchased from Sigma (Milan, Italy). Solutions of the above-mentioned compounds were prepared according to Sticozzi et al. (2013).

### Cells

Human glioblastoma astrocytoma U-373 MG cells (U-373 MG, Sigma catalogue number 89081403) were cultured in Dulbecco’s modified Eagle’s medium (DMEM), supplemented with 10% fetal calf serum (FCS), 100 U/ml penicillin, 100 μg/ml streptomycin and 2 mM L-glutamine, and incubated at 37 °C for 24 h in 95% air/5% CO_2_ until 80% confluence. Before each treatment, cells were left in fresh serum-free medium for 12 h.

### Intracellular Calcium Concentration

The method used has been described previously (Sticozzi et al. 2013). Briefly, U-373 MG cells were stimulated for 0, 15, 30, 45, 60 and 75 min with different cytokines. At the end of each time point, cells were washed and incubated for 30 min with 1 μM Fluo-3 acetoxymethyl ester (Fluo3/AM) dissolved in Hanks' balanced salt solution (HBSS) (in mM: NaCl, 145; KCl, 5; HEPES, 10; glucose, 10; MgCl, 1; CaCl_2_, 1,3) supplemented with 0.03% pluronic F-127. After washing of cells, calcium fluorescence was recorded at 485 nm and 538 nm emission wavelengths (Thermo Scientific Appliskan, M-Medical, Milan, Italy). Fluorescence intensity changes in the calcium indicator Fluo-3 (ΔDF) were determined relative to baseline fluorescence in control cells (*F*_0_) under identical experimental conditions at corresponding time points, and the ratio of ΔF/F_0_ was determined. Each sample was calibrated to evaluate [Ca^2+^]. To obtain maximum fluorescence (*F*max), 2.3 μM ionomycin was added, followed by 20 mM MnCl_2_ to record the autofluorescence of the system. Intracellular Ca^2+^ values were obtained from the observed fluorescence (Δ*F*) after correction of *F*, *F*_max_ and *F*_min_ for autofluorescence and F_0_. For each well, the intracellular calcium concentration ([Ca^2+^]_i_) was calculated according to the following formula: [Ca^2+^]_i_ = (F − F_min_)(F_max_ − F) − ^1^ x *K*_d_, where *K*_d_ is the dissociation constant of Fluo-3 (390 nM), F is the observed fluorescence, F_max_ the fluorescence at high [Ca^2+^] and F_min_ is the fluorescence of the Ca^2+^ free indicator. The latter correspond to [(F_max_ − C) × 0.025], where C is the autofluorescence.

### Determination of cGMP

The assessment of cGMP levels in U-373 MG cells was performed with an enzyme immunoassay kit (Cayman, Italy) according to the manufacturer’s instructions. Cells were stimulated for 15, 30 and 60 min with IL-6 or TNF-α or pretreated with 1400 W for 30 min before being treated as previously described. Proteins were measured using a commercially available Bradford method-based assay kit (Bio-Rad protein assay, Milan, Italy).

### GFAP Immunocytochemistry

U-373 MG cells (2 × 10 ^4^ cells/ml, grown on glass coverslips) were stimulated for 1 h with IL-6 or TNF-α alone or in combination with inhibitors in serum-free medium. After further incubation in culture medium supplemented with 1% FCS for different durations, cells were fixed using ice-cold 4% paraformaldehyde (40 min, room temperature). After three washes in phosphate-buffered saline (PBS), 0.02% Triton X-100 was used to permeabilize the cells, while nonspecific binding sites were blocked by 1% bovine serum albumin (BSA)-PBS (45 min, room temperature). The slides were then treated with anti-GFAP primary antibodies (1:20, 1 h at room temperature) and then with FITC-conjugated anti-mouse secondary antibodies (1:100, 30 min). Negative controls were prepared by omitting primary antibodies. Slides were mounted with DABCO antifade mounting medium (1,4-diazabicyclo[2.2.2]octane) in glycerine and analysed using a Zeiss Axioplan 2 light microscope equipped with epifluorescence coupled with AxioVision version 4.6.3 software, according to Sticozzi et al. (2013). GFAP was semi-quantified as immunoreactivity area (μm^2^) normalized to the total cellular area (DAPI staining) and expressed as a percentage of controls. At least five fields/coverslip over a total of 8–10 coverslips/experiment were analysed.

### Inducible nitric oxide synthase (iNOS) Western Blot

After stimulation with IL-6 or TNF-α (1 h), U-373 MG cells were treated with lysis buffer (20 mM Tris base, pH 7.4, 75 mM NaCl, 20 mM ethylene glycol-bis(β-aminoethyl ether)-*N*,*N*,*N*′,*N*′-tetraacetic acid [EGTA], 1 mM Na_3_VO_4_, 2.5 mM NaF, 2.5 μg/mL aprotinin, 1 mM phenylmethylsulfonyl fluoride, 10 μg/ml leupeptin, 1 mM sodium pyrophosphate and 1% Triton X-100), frozen at −80 °C for 24 h and defrosted on ice. A total of 50–60 μg protein (Bio-Rad protein assay, Milan, Italy) in 3× loading buffer (65 mM Tris base, pH 7.4, 20% glycerol, 2% sodium dodecyl sulphate, 5% β-mercaptoethanol and 1% bromophenol blue) was boiled for 5 min, loaded onto 10% sodium dodecyl sulphate-polyacrylamide electrophoresis gels and separated by molecular size. The gels were then electro-blotted onto nitrocellulose membranes for 1 h at 450 mA at 100 mV in 0.025 M Tris, 0.192 M glycine, 0.1% SDS and 20% methanol. The blots were blocked with Tris-buffered saline (TBS) (0.05 M Tris-HC1, pH 7.4, 0.15 M NaCl), containing 1% BSA and 0.1% Tween 20, for 2 h and incubated with primary antibody (rabbit anti-iNOS, 1:1000, overnight at 4 °C). The blots were washed and incubated with horseradish peroxidase (HRP)-conjugated secondary antibody (anti-rabbit, 1:3000, 2 h at room temperature), and the protein was visualized by autoradiography on film using an enzyme-linked chemiluminescence technique. The blots were then stripped and re-probed with β-tubulin (1:1000) to assess the total protein load.

### Animals

The procedures used complied with European legislation on the use and care of laboratory animals (EU Directive 2010/63) and National Institutes of Health guidelines. Male albino Sprague Dawley rats weighing 300 ± 50 g were purchased from Charles River (Italy, www.criver.com). Two to four rats per cage were housed in a room maintained at constant temperature (23–24 °C) and humidity (50–60%), on a 12 h light/dark cycle, with free access to food and water*.*

### Rat Striatal Brain Slices and ^45^Ca^2+^ Release Assay

**Fig. 1:**
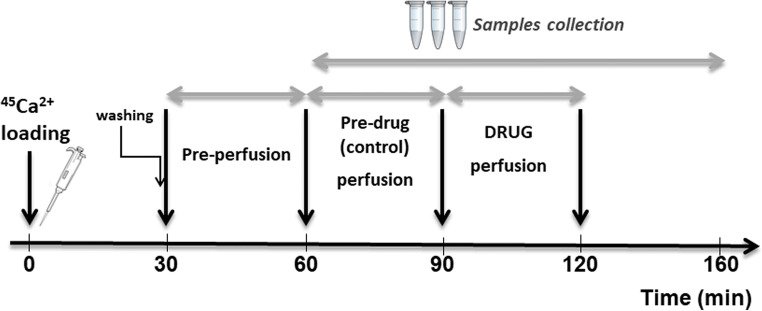
Overview of the experimental design

Striatal slices were prepared following a method described previously (Meini et al. 2000, 2003; Plami et al. 1996). Briefly, 350 μm striatum slices were incubated with 1 ml of PSS containing 4 μCi of ^45^Ca^2+^ (specific activity 29.46 mCi/mg, 3.0 μM) at 37 °C under 95% O_2_–5% CO_2_ continuous gentle bubbling for 30 min (Plami et al. 1996). After washes in PSS, batches of 3–5 slices were placed in micro-perfusion chambers and super-fused (0.5 ml/min, 37 °C) with oxygenated Ca-EGTA-buffered PSS. After the pre-perfusion period during which ^45^Ca^2+^ release stabilized (25 min, see Fig. 1), fluid was collected continuously (3 min samples, 1.5 ml) for 100 min. Spontaneous calcium release from striatal slices under conditions of no stimulation was constant over the sample collection period (100 min). Radioactivity in each sample, along with that remaining in tissues, was determined, and the release of ^45^Ca^2+^ was expressed as the percentage of residual radioactivity in the tissue at each sampling interval [fractional release (FR)] (Plami et al. 1996).

### In Vivo Experiments

Under anaesthesia (Ketavet^®^ 60 mg/kg and Rompun^®^, 16 mg/kg body weight), the rat striatum was injected with IL-6, TNF-α or diethylenetriamine (DETA)/NO dissolved in 5 μl of endotoxin-free saline using a Hamilton syringe. The following stereotaxic coordinates were used: anteroposterior (AP), −0.3 mm; mediolateral (ML), −3 mm; dorsoventral (DV), −5 mm. At different time points, rats were then perfused transcardially with heparinized saline and decerebrated. The removed brain was frozen in liquid nitrogen and stored at −80 °C until use (Sticozzi et al. 2013).

### Immunohistochemistry

Brain sections taken from the lesion tip area were fixed for 20 min in ice-cold acetone at 4 °C. After three washes in PBS, brain sections were incubated in blocking solution (5% normal goat serum (NGS)-PBS in 0.3% Triton X-100, 1 h at room temperature). The sections were then treated with anti-GFAP primary antibody (1:100, 1 h at room temperature), incubated with FITC-anti-mouse secondary antibody (1:100, 1 h at room temperature), rinsed in PBS and counterstained with DAPI. Samples were mounted and examined using a Leica (LEITZ DMRB) light microscope equipped with epifluorescence. Z-stack images (no. steps: ~20; Z-step size: ~1 μm; Z-volume: ~19) were acquired using the Leica Application Suite Advanced Fluorescence 1.8.0 build 1346 (AF6000_DFC) and analysed using Leica microsystem LAS AF-6000 software. GFAP was semi-quantified as immunoreactivity area (mm^2^) normalized to the total number of cells and expressed as a percentage of controls (animals injected with endotoxin-free saline).

## Western Blot Analysis

Brain cryosections were defrosted, and the striatum was excised and homogenized in lysis buffer. After centrifugation, 50–60 μg protein of the supernatant in 3× loading buffer was boiled, loaded onto SDS and electro-blotted. Membranes were incubated with primary antibody (mouse anti-GFAP, overnight at 4 °C). The blots were washed and incubated with HRP-conjugated secondary antibody (anti-mouse, 1:5000, 2 h at room temperature), and the protein was visualized by autoradiography on film using an enzyme-linked chemiluminescence technique. The blots were then stripped and re-probed with β-tubulin (1:1000) to assess the total protein load.

### Determination of cGMP in Brain

Cryosections were defrosted, and the striatum was excised and homogenized in trichloroacetic acid. After brief centrifugation, the cGMP levels in supernatant were determined as described above.

### Statistical Analysis

Data are reported as mean ± SEM from at least 4–5 (cells) or 3–4 (rats) independent experiments. Comparison was performed by analysis of variance (ANOVA) followed by Bonferroni post-test (GraphPad Prism version 5.04, GraphPad Software Inc., San Diego, CA, USA). In all comparisons, the level of statistical significance (*p*) was set at 0.05.

## Results

### Human Glioblastoma Astrocytoma U-373 MG Cells

#### IL-6 and TNF-α, but Not IL-4 and IL-10, Increase GFAP Expression

**Fig. 2:**
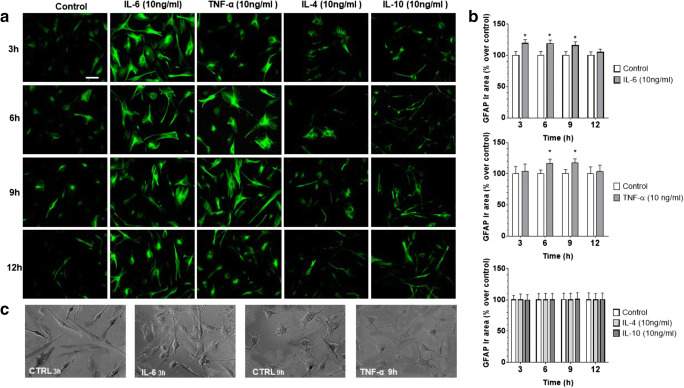
IL-6 and TNF-α, but not IL-4 and IL-10, upregulate GFAP expression in human glioblastoma astrocytoma U-373 MG cells. (**a**) Cells were stimulated for 1 h with IL-6, TNF-α, IL-4 or IL-10 (all tested at 10 ng/ml), and 3, 6, 9 and 12 h later, immunostained for GFAP (scale bar: 50 μm) (original magnification ×200). (**b**) GFAP was semi-quantified as immunoreactivity area (μm^2^), normalized to the total number of cells and expressed as percentage of untreated cells (control). Values are the means ± SEM of five microscopic fields per coverslip from a total of 8–10 coverslips. *p < 0.05 and **p < 0.01 vs control, same time (ANOVA and Bonferroni post-test). (**c**) Phase contrast showing changes in astrocytic profile due to treatments which include cellular hypertrophy, increased branched processes and synaptic connectivity (scale bar: 50 μm)

To establish the role of IL-6, TNF-α, IL-4 and IL-10 in human glioblastoma astrocytoma U-373 MG cell reactivity, the effects of these cytokines on GFAP expression were investigated. All cytokines were tested at 10 ng/ml. As shown by immunocytochemistry imaging and by semi-quantified analysis of the immunoreactivity area (Fig. 2), after IL-6 stimulation, GFAP peaked already at 3 h, remained constant at 6 h and 9 h, and regained control values at 12 h. Similarly, TNF-α promoted an increase in GFAP, which was however delayed compared to that of IL-6, as it peaked significantly at 6–9 h stimulation to decline at 12 h. For this reason, 3 h and 9 h were then taken as specific time points for IL-6 and TNF-α GFAP stimulation, respectively. Phase contrast analysis showed changes in astrocytic morphology upon treatment, characterized by cellular hypertrophy, and increased branched processes and synaptic connectivity. Finally, in contrast to pro-inflammatory cytokines, neither IL-4 nor IL-10 had effects on GFAP.

### NO/cGMP and Intracellular Calcium Stores Are Involved in IL-6- and TNF-α-Induced GFAP Expression

**Fig. 3:**
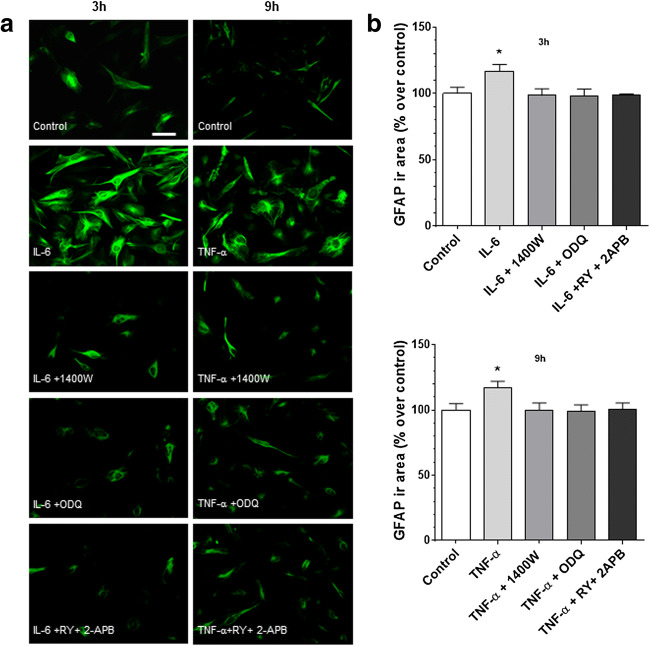
NO, cGMP and ryanodine plus inositol-(1,4,5)-trisphosphate-sensitive calcium stores mediate IL-6- and TNF-α-induced increase of GFAP expression in human glioblastoma astrocytoma U-373 MG cells. (**a**) Cells are pretreated for 30 min with 1400 W (1 μM), ODQ (5 μM) or RY (10 μM) plus 2APB (100 μM) before being stimulated for 1 h with IL-6 or TNF-α (both 10 ng/ml). After these treatments (3 and 9 h, respectively), cells were immunostained for GFAP (scale bar: 50 μm) (original magnification ×200). (**b**) GFAP is semi-quantified as immunoreactivity area (μm^2^) normalized to the total number of cells and expressed as percentage of untreated cells (control). Values are the means ± SEM of five microscopic fields per coverslip from a total of 8–10 coverslips. *p < 0.05 vs control (ANOVA and Bonferroni post-test)

To verify whether NO/cGMP were part of the intracellular signalling responsible for increased GFAP expression induced by IL-6 or TNF-α, cells were pretreated for 30 min with the selective iNOS and soluble guanylate cyclase (sGC) inhibitors (1400 W 10 μM, and ODQ 100 μM, respectively). Human glioblastoma astrocytoma U-373 MG cells were then stimulated with IL-6 (10 ng/ml, 3 h) or TNF-α (10 ng/ml, 9 h). Interestingly, both inhibitors prevented an increase in GFAP elicited by both IL-6 and TNF-α (Fig. 3), while 1400 W and ODQ did not affect GFAP response per se (data not shown).

To determine whether stored calcium was involved in the expression of GFAP, the effects of combined administration of IP3- and RY-sensitive receptors inhibitors, 2APB and RY respectively, were investigated. As shown in Fig. 3, 30 min RY (10 μM) plus 2APB (100 μM) pretreatment reversed the effects elicited by the cytokines. Taken together, these results suggest that the IL-6- and TNF-α-dependent upregulation of GFAP expression is mediated by NO/cGMP/Ca^2+^ signalling and that the intracellular calcium stores are involved.

### IL-6 and TNF-α, but Not IL-4 and IL-10, Affect Intracellular Calcium Concentration and iNOS Expression

**Fig. 4:**
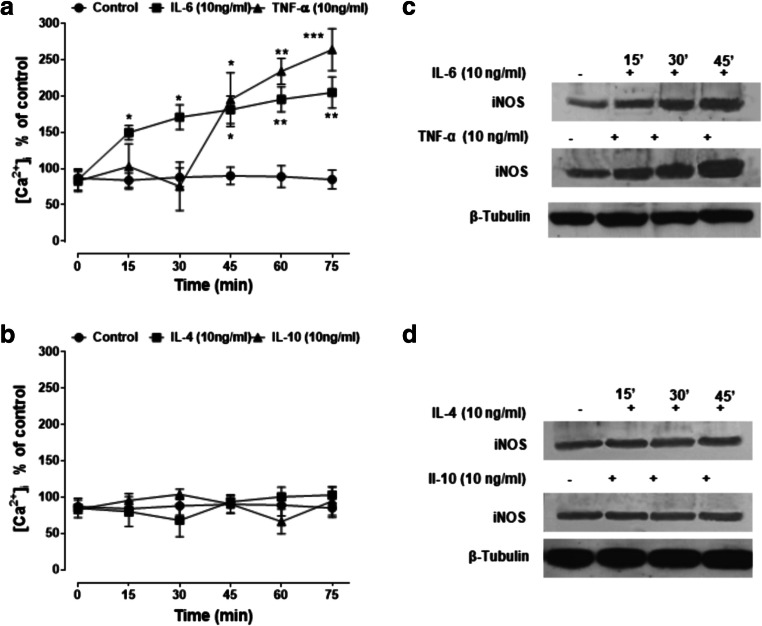
IL-6 and TNF-α, but not IL-4 and IL-10, increase intracellular calcium concentration and iNOS expression in human glioblastoma astrocytoma U-373 MG cells. Human glioblastoma astrocytoma U-373 MG cells were stimulated for different durations (0–75 min) with IL-6 or TNF-α (**a**) and IL-4 or IL-10 (**b**). All cytokines were tested at 10 ng/ml. Intracellular calcium and iNOS were determined by Fluo-3 fluorometric and Western blot analysis, respectively. For [Ca^2+^]_i_, values are means ± SEM; *p < 0.05 and **p < 0.01 vs matched-time controls (ANOVA and Bonferroni post-test). (**c**, **d**) Representative Western blot images of iNOS expression taken from a total of five independent experiments

Over the stimulation period of 75 min, both IL-6 and TNF-α increased [Ca^2+^]_i_ by two to three times the basal levels (50–100 nM) (Fig. 4a). IL-6 had a more immediate effect than TNF-α, as [Ca^2+^]_i_ was increased already within 15 min of stimulation and then continued to rise slowly, while the effect of TNF-α was delayed by about 30 min, rising sharply thereafter. In contrast to pro-inflammatory cytokines, both IL-4 and IL-10 failed to elicit the boosting effects on [Ca^2+^]_i_ (Fig. 4b). Interestingly, iNOS expression increased progressively during the 45 min stimulation with both cytokines (Fig. 4c). The enzyme, however, was also found in untreated samples, indicating that it is constitutively expressed in U-373 MG cells. Both IL-4 and IL-10 failed to upregulate iNOS expression (Fig. 4d).

**Fig. 5:**
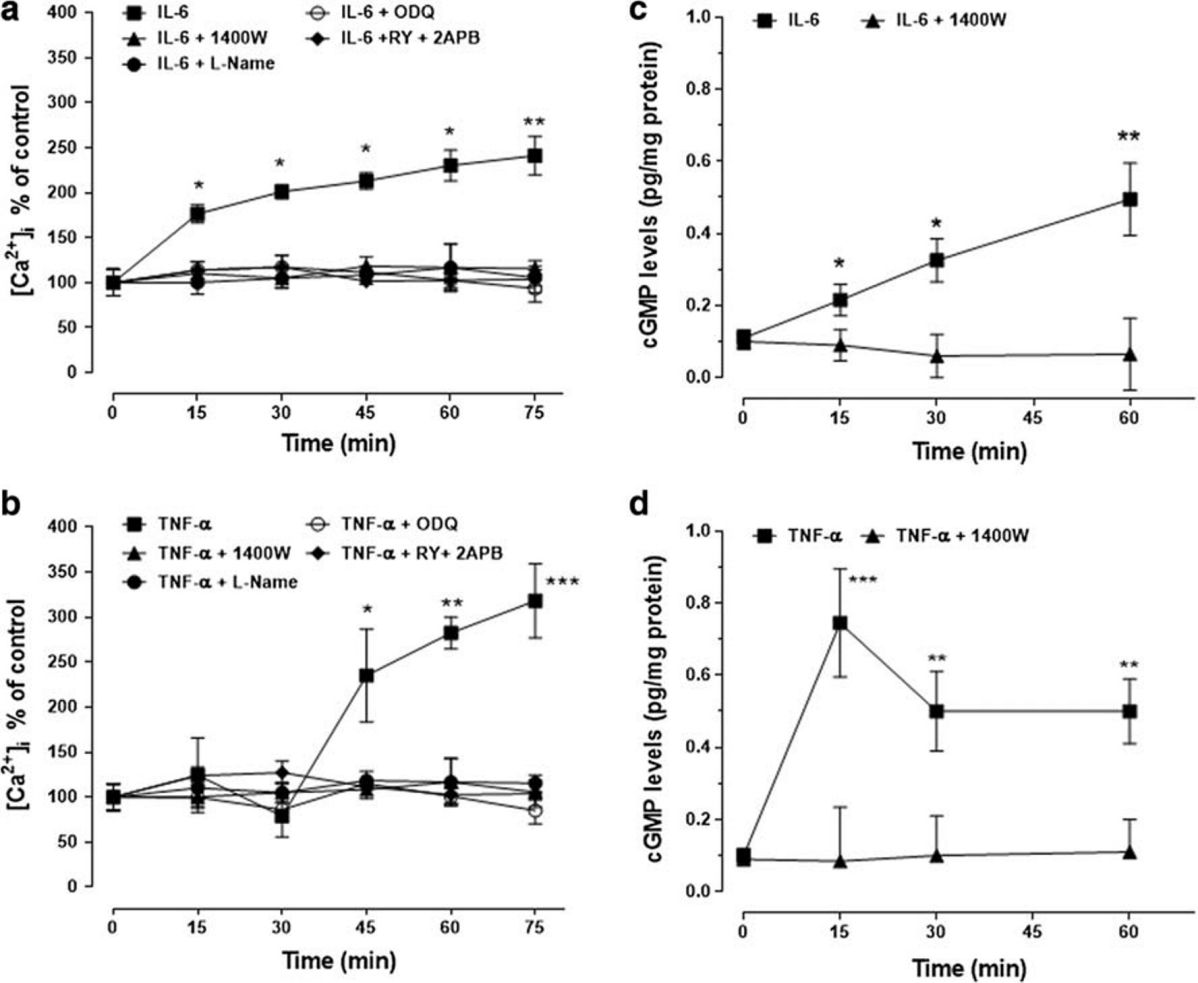
NO/cGMP and ryanodine- plus inositol-(1,4,5)-trisphosphate-sensitive calcium stores mediate IL-6- and TNF-α-induced increase in intracellular calcium concentrations in human glioblastoma astrocytoma U-373 MG cells. (**a**, **b**) Cells were pretreated for 30 min with 1400 W (1 μM), ODQ (100 μM) or RY (10 μM) plus 2APB (100 μM) before stimulation with IL-6 or TNF-α for different periods. (**c**, **d**) Cells were stimulated with the same cytokines in the presence (30 min pretreatment) or absence of 1400 W (10 μM). Values are means ± SEM; *p < 0.05 and **p < 0.01, ***p < 0.001 vs matched-time controls (ANOVA and Bonferroni post-test)

To further confirm the involvement of [Ca^2+^]_i_ and NO/cGMP signalling, human glioblastoma astrocytoma U373 cells were pretreated for 30 min with iNOS or sGC inhibitors, or RY plus 2APB, before stimulation with IL-6 (Fig. 5a) or TNF-α (Fig. 5b) for different durations. Both 1400 W (10 μM) and L-NAME (3 mM) prevented the boosting effect of IL-6 and TNF-α on calcium responses. The same effect was reproduced by ODQ (100 μM) and RY (10 μM) plus 2APB (100 μM). As NO activates soluble guanylate cyclase with consequent accumulation of cGMP (Monti et al. 2010), cGMP itself was assessed as an indirect measure of NO production. Results showed that IL-6 (Fig. 5c) and TNF-α (Fig. 5d) indeed increased cGMP levels, as prevented by 1400 W.

### Rat Brain Striatum: Ex Vivo Study

#### Effects of IL-6 and TNF-α on ^45^Ca^2+^ Release: Role of NO and cGMP

**Fig. 6:**
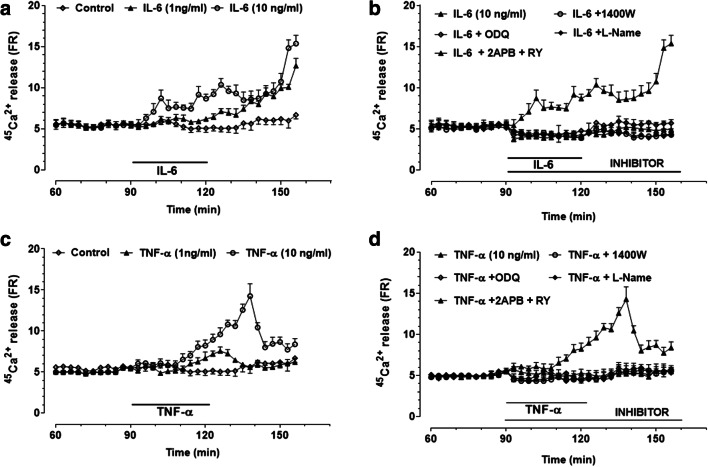
IL-6- and TNF-α-induced calcium efflux from rat striatal slices is mediated by NO, cGMP and ryanodine (RY)- plus inositol-(1,4,5)-trisphosphate (IP_3_)-sensitive calcium stores. (**a**) ^45^Ca^2+^ efflux in the presence of IL-6 (1 ng/ml and 10 ng/ml). (**b**) ^45^Ca^2+^ efflux in the presence of IL-6 (10 ng/ml) and selective inhibitors of the inducible NO synthase (1400 W), guanylate cyclase (ODQ) and RY- plus IP_3_-sensitive calcium channels (2APB) and the NOS inhibitor L-NAME. (**c**) ^45^Ca^2+^ efflux in the presence of TNF-α (1 ng/ml and 10 ng/ml). (**d**) ^45^Ca^2+^ efflux in the presence of TNF-α (10 ng/ml) and selective inhibitors of the inducible NO synthase (1400 W), guanylate cyclase (ODQ) and RY- plus IP_3_-sensitive calcium channels (2APB) and the NOS inhibitor L-NAME. ^45^Ca^2+^release was expressed as a percentage of the residual radioactivity present in the tissue at each sampling interval (FR; see Materials and Methods). Values are the means ± SEM. Selective inhibitors were used at the following concentrations: 1400 W (10 μM), ODQ (100 μM), RY (10 μM) plus 2APB (100 μM) and L-NAME (3mM)

IL-6 (1 ng/ ml) induced a slow increase in calcium efflux that continued to increase progressively even after cytokine washout, peaking at the end of the perfusion period (Fig. 6a). This effect was concentration-dependent, as 10 ng/ml IL-6 potentiated and shortened the onset of the response. To establish whether NO production was involved in the intracellular signalling responsible for IL-6-induced calcium release, slices were pretreated with 1400 W (10 μM) and L-NAME (3 mM). The former inhibitor, which did not affect spontaneous ^45^Ca^2+^ efflux per se (data not shown), completely reversed the ^45^Ca^2+^ release induced by 10 ng/ml IL-6 (Fig. 6b). The finding that L-NAME elicited an effect like that of 1400 W suggests that both the inducible and the constitutive forms of NOS were involved. To further verify whether cGMP mediated the effect of NO on ^45^Ca^2+^ efflux, slices were pretreated with ODQ (100 μM). As shown in Fig. 6b, this was the case, as this compound completely reversed the effect of IL-6 on calcium response, being ineffective per se (data not shown). Moreover, the combined administration of 2APB (100 μM) and RY (10 μM) completely prevented the increase in ^45^Ca^2+^ release induced by the cytokine, reducing the response to baseline values (Fig. 6b). ^45^Ca^2+^ release was unaffected by 2APB plus RY alone (data not shown). When the effects of TNF-α (1 and 10 ng/ml) were investigated, it was found that this cytokine also promoted a concentration-dependent increase in ^45^Ca^2+^ efflux (Fig. 6c), which was however more transient than that of IL-6. To verify whether NO, cGMP and calcium stores were also involved in this response, TNF-α was perfused in combination with the above-mentioned inhibitors. In the presence of 1400 W or L-NAME, the boosting effect of TNF-α on ^45^Ca^2+^ release was reduced to control values. Similar results were observed with ODQ or RY plus 2APB (Fig. 6d).

### Rat Brain Striatum: In Vivo Study

#### Effects of IL-6, TNF-α and DETA/NO on cGMP and GFAP Expression

**Fig. 7:**
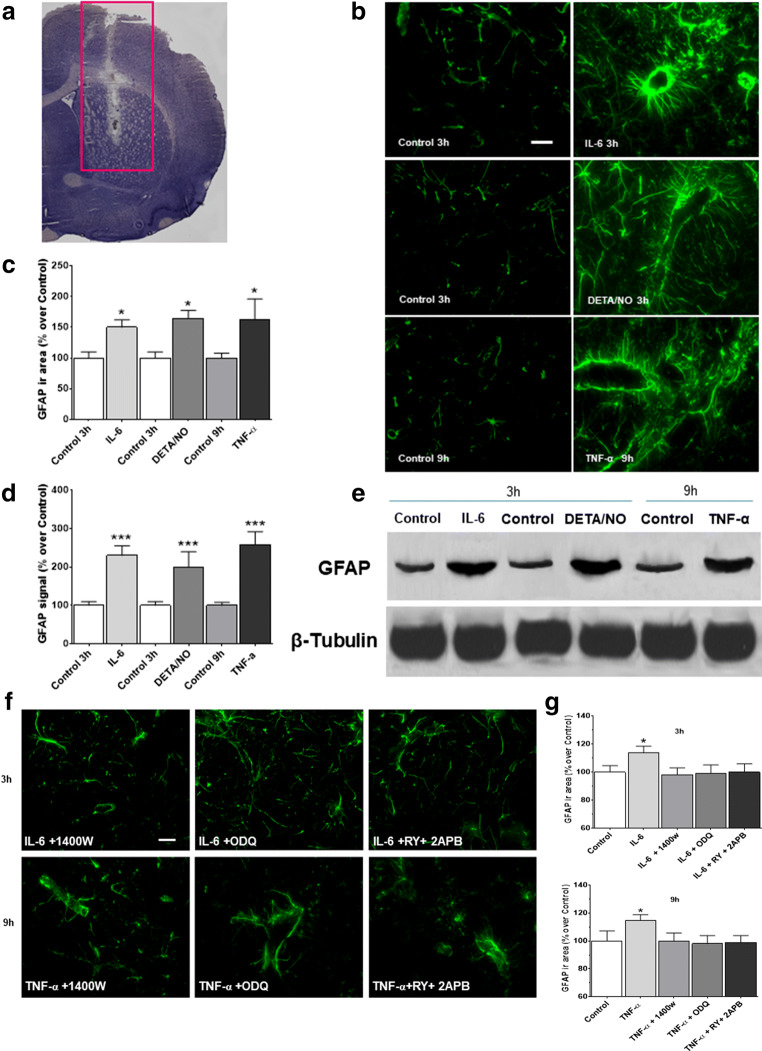
IL-6, TNF-α and DETA/NO upregulate GFAP expression in rat brain striatum, an effect mediated by NO, cGMP and ryanodine (RY)- plus inositol-(1,4,5)-trisphosphate (IP3)-sensitive calcium stores. IL-6 (50 ng), TNF-α (50 ng) or DETA/NO (1 mM) was injected into the brain striatum of anaesthetised rats. At 3, 9 and 3 h after these treatments, respectively, the brain was removed, frozen and serially cryosectioned. One series of these sections (see Methods) was used for GFAP immunofluorescence, while another was defrosted, and the striatum excised, homogenized, and used for Western blot analysis for the same protein. (**a**) Toluidine blue-stained tissue shows the analysed site (red rectangle) (**b**) A representative experiment of GFAP immunofluorescence shows a particularly intense signal around the blood vessels, likely representing blood–brain barrier astrocytes (scale bar: 50 μM) (original magnification ×200). (**c**) Semi-quantitative analysis of GFAP immunofluorescence, expressed as percentage of controls (0 h sham-operated animals), is reported as the mean ± SEM. (**d**) Semi-quantitative analysis of GFAP immunoblot spots, normalized to β-tubulin and expressed as percentage of controls, and (**e**) a representative GFAP immunoblot. (**f**) A representative experiment of GFAP immunofluorescence after 1400 W (10 μM), ODQ (100 μM) and RY (10 μM) plus 2APB (100 μM) pretreatment. (**g**) Semi-quantitative analysis of GFAP immunofluorescence, expressed as a percentage of controls (0 h sham-operated animals, i.e. controls, CTRL). Panels (c), (d) and (g): data are reported as mean ± SEM. *p < 0.05, ***p < 0.01 vs matched-time controls (ANOVA and Bonferroni post-test)

The effects of IL-6 and TNF-α on GFAP expression were studied in vivo following cytokine administration in the brain striatum of anaesthetised rats (Fig. 7). Results showed that the expression of GFAP increased 3 and 9 h after stimulation with IL-6 (50 ng) and TNF-α (50 ng), respectively (Fig. 7b-c), in agreement with in vitro data. Interestingly, the NO donor DETA/NO (1 mM) mimicked the effects of the cytokines on the GFAP response, as also confirmed by Western blot analysis (Fig. 7d-e). To determine whether NO, cGMP and calcium stores are involved in this response, IL-6 or TNF-α was injected in combination with 1400 W (10 μM) ODQ (100 μM) and RY (10 μM) plus 2APB (100 μM). In the presence of 1400 W, the TNF-α-mediated increase in GFAP expression was reversed; similar results were observed with ODQ or RY plus 2APB (Fig. 7f–g). Finally, cGMP was increased by 300–500% over control values 15 and 30 min after the administration of IL-6 or TNF- α in the striatum (Table. 1). Taken together, these findings corroborate previous data and represent direct evidence of the involvement of NO/cGMP in the in vivo signalling cascade leading to astrocyte activation.

**Table 1:** IL-6, TNF-α and DETA/NO upregulate cGMP levels in rat brain striatum

	cGMP (pg/mg protein)	
	15 min	30 min
Control	0.80 ± 0.06	0.66 ± 0.02
IL-6 (50 ng)	2.86 ± 0.28**	2.53 ± 0.21**
DETA/NO (1 mM)	2.63 ± 0.25**	2.73 ± 0.29**
TNF-α (50 ng)	3.80 ± 0.30***	2.63 ± 0.23**

Values represent the means ± SEM. ** *p* < 0.01, *** *p* < 0.001 vs control (0 h sham-operated animals) (ANOVA and Bonferroni post-test)

## Discussion

After CNS injury, astrocytes transition to a reactive state undergoing morphological and molecular changes, characterized by increased expression of proteins including GFAP, vimentin, nestin and iNOS (Lagos-Cabre et al. 2017). Modulators of GFAP expression include steroids, cytokines and growth factors (Laping et al. 1994; John et al. 2003; Lagos-Cabre et al. 2019). The observation that NO/cGMP-dependent calcium signalling mediates IL-1β-induced astrocyte proliferation (Meini et al. 2008; Sticozzi et al. 2013), together with the in vivo evidence of IL-6-induced astrogliosis (Woiciechowsky et al. 2004), prompted us to investigate the effects of IL-6 and TNF-α on GFAP expression and the role of NO/cGMP-dependent calcium signalling in this response. The effects were compared to that induced by the anti-inflammatory cytokines IL-4 and IL-10. Results highlighted that in human glioblastoma astrocytoma U-373 MG cells, IL-6 and TNF-α, but not IL-4 and IL-10, increased GFAP expression, increased intracellular calcium with kinetics comparable to those in tissues, and increased iNOS expression and cGMP. These effects were reversed by 1400 W, ODQ and RY plus 2APB, thus suggesting that IL-6- and TNF-α-dependent upregulation of GFAP expression is mediated by NO/cGMP/calcium signalling and that intracellular calcium stores are involved. Interestingly, IL-6 and TNF-α have been proven to activate iNOS in adult rat ventricular cardiomyocytes (Vasquez-Vivar et al. 2008), C57BL/6 mice bone marrow stromal cells (Xu et al. 2008) and mouse microvascular endothelial cells (Zhou et al. 2008), thus corroborating our findings. Moreover, as iNOS is constitutively expressed in U373-MG cells and increases early after different treatments (Nanetti et al. 2005 and the present results), it can release NO over an interval compatible with the calcium responses. The concurrent presence of neuronal nitric oxide synthase (nNOS) in U-373 MG cells (Nanetti et al. 2005), however, might suggest a possible role of this isoform as well. As the selective iNOS inhibitor 1400 W completely reversed the iNOS-mediated effects, this rules out the possible involvement of nNOS. Taken together, the results obtained in U-373 MG cells indicated that iNOS/cGMP-dependent calcium signalling mediates the IL-6- and TNF-α-induced increase in GFAP expression.

The effects of the above-mentioned cytokines were then further investigated in a tissue context, i.e. striatal slices, which maintain functional local synaptic circuitry and preserve brain architecture. Results showed that IL-6 and TNF-α promoted an increase in calcium efflux, reversed by the selective nitric oxide synthase and guanylate cyclase inhibitors, suggesting the involvement of NO/cGMP production. Furthermore, as the same calcium responses were counteracted by combined treatment with ryanodine and 2APB, the possibility of RY- and IP_3_-sensitive intracellular calcium stores being the main sources of the released calcium might be advanced.

It is well established that many NO responses in different tissues and cells are elicited via activation of soluble guanylate cyclase and cGMP generation. NO-mediated activation of a G kinase is generally accepted as part of the overall mechanism (Fiscus 2002), although in most cases the precise signalling pathways are still under investigation. Interestingly, the NO/guanylate cyclase/cGMP pathway was found to regulate the expression of GFAP in astrocytes via a protein kinase G (PKG) (Brahmachari et al. 2006) and to alter cytoskeletal dynamics (Boran and Garcia 2007), thus supporting the present findings.

The kinetics of the calcium changes, however, were different according to the cytokine analysed: in cells, TNF-α generated a much greater release than IL-6, which instead induced a progressive and slower rise. This might reflect differences in their relative effects on intracellular calcium. Indeed, as in tissue experiments, IL-6 induced a prompt increase in intracellular calcium soon after its administration, whereas an equivalent concentration of TNF-α gave rise to delayed (15–30 min) and reduced response. This might be explained in view of our previous findings showing that the amount of NO is crucial in triggering the calcium response in striatal tissue, as a moderate amount stimulates, while excessive NO inhibits, calcium release (Meini et al. 2000, 2003). Different NO donors such as DEA/NO and Sper/NO, in fact, release it with different kinetics, and the resulting calcium responses are inversely correlated with the amounts of NO generated (Meini et al. 2000). Thus, in the present study, the lag phase in calcium response observed with TNF-α might reflect the progressive decrease in NO concentration which were inibitory at the beginning. These results are also consistent with literature data showing NO as a pathophysiological modulator of apoptosis (Brookes et al. 2000), cell proliferation and cell cycle arrest (Napoli et al. 2013), inflammation, angiogenesis and cancer (Vasudevan and Thomas 2014) depending on its concentration.

Accumulating evidence indicates that cGMP enhances the production of cyclic ADP ribose, which in turn induces the release of calcium from caffeine/ryanodine stores (Higashida et al. 2001). Therefore, it is likely that NO/cGMP signalling is the mechanism triggering the increased calcium efflux induced by IL-6 and TNF-α, probably via synthesis of cyclic ADP ribose, even though the precise cellular target remains to be elucidated. Furthermore, increased calcium release from striatal slices induced by IL-1β is partly dependent on extracellular calcium via “calcium-induced calcium release” (Meini et al. 2000, 2003), and in line with this hypothesis, other authors have also shown that both store- and receptor-operated calcium entry are involved in IL-1β-induced calcium signals (Beskina et al. 2007; Sama et al. 2008). Thus, it is tempting to speculate that the source of calcium changes induced by IL-6 and TNF-α might depend on similar mechanism(s).

To further assess whether NO/cGMP/calcium signalling was involved in promoting astrocyte activation, in vivo investigations were also performed. These results substantiated those in cells and in the striatum, as both IL-6 and TNF-α upregulated GFAP expression and increased levels of NO-dependent cGMP. These effects were mimicked by DETA/NO, a compound that releases NO.

Different aspects of astroglial calcium homeostasis have been shown to be regulated by NO via cGMP (Matyash et al. 2001; Willmott et al. 2000), and important properties of the main pro-inflammatory cytokines including IL-6, TNF-α and IL-1β are mediated by this signalling. Therefore, while adding a new functional role in the regulation of astrocyte reactivity, our findings indicate that this mechanism may be part of a more general process subserving cytokine-induced inflammation.

The likelihood that peripheral and/or central immune-like cells other than astrocytes were sensitive to the same signalling cannot be ruled out. Indeed, both activated microglia, a major source of NO, and Th1-differentiated lymphocytes, consistent features of brain inflammation, release IL-1, IL-2, IL-6 and TNF-α, which in turn further contribute to glia activation, progression of inflammation and eventually cytotoxicity by an autocrine and paracrine process (Block and Hong 2007; Wei et al. 2014; Greenhalgh et al. 2020; Illes et al. 2020). Increasing evidence also suggests that crosstalk among different cell types and cytokines released by them is involved in downregulation of astrocyte reactivity and amelioration of inflammation (Sofroniew 2015). Indeed, IL-4 and IL-10, a product of macrophages, microglia and lymphocytes, once generated, inhibit the release of pro-inflammatory cytokines from the same cells, resulting in indirect downregulation of astrocyte reactivity (Harry and Kraft 2008). Moreover, alterations in brain-derived IL-2 and IL-2 receptors, potentially produced by neurons and astrocytes (Shen et al. 2010), have been implicated in the pathogenesis of several major neurological disorders due to their immunoregulatory functions (Wei et al. 2014); among these, the ability to drive GFAP+ astrocyte-targeted production of IL-10, which in turn modulates the numbers of microglia/macrophages, has been postulated (Recasens et al. 2019). Therefore, it would be interesting to investigate whether opposite regulation of the identified signalling by functionally different cytokines might be involved in modulating astrocyte reactivity, especially when considering that neuroinflammation associated with neurodegenerative diseases (AD, PD, multiple sclerosis, amyotrophic lateral sclerosis and ischemia) constitutes a superimposed exacerbating factor.

Finally, although controversial, moderate reactive astrogliosis may be crucial for recovery following CNS injury, due to secretion of neurotrophic factors, while rapid, severe and prolonged activity of these cells is believed to augment or initiate a massive inflammatory response leading to neuronal death, as observed in chronic neurodegenerative diseases (Zhang et al. 2010). On the other hand, emerging evidence indicates that GFAP, far from being a simple marker of astrogliosis, plays an important physiological and pathophysiological role in the CNS, as it controls the shape and movement of astrocytes, their interaction with neurons, the modulation of synaptic efficacy, blood–brain barrier integrity and CNS myelination (Brahmachari et al. 2006; Hol and Pekny 2015; Liddelow and Barres 2017). In contrast, the extent of GFAP expression seems to correspond to the severity of astroglial activation and neurodegeneration in various neuroinflammatory-based disorders, including Alzheimer’s disease, Parkinson’s disease, HIV dementia and multiple sclerosis (Sadick and Liddelow 2019). Interestingly, the present findings highlight that GFAP expression was increased via a NO/cGMP/calcium pathway after cytokine administration, suggesting that targeting this signalling might be a tool for regulating GFAP expression or astrogliosis, thus contributing to a therapeutic approach for neuroinflammatory disorders.

## Conclusion

Moderate astrogliosis may have beneficial effects in the amelioration of CNS injury, while massive activation, and the subsequent impairment of neuronal function, characterizes several neurodegenerative disorders. Interestingly, in Alzheimer’s and Parkinson’s diseases, increased GFAP expression seems to correlate with the severity of astroglial activation (Laurent et al. 2018; Kanthasamy et al. 2019). Furthermore, increasing evidence indicates that GFAP itself contributes to the promotion of inflammation and neurotoxicity (Brahmachari et al. 2006). The present results demonstrate that NO/cGMP-dependent intrastore calcium mobilization mediates TNF-α- and IL-6-induced GFAP expression in vitro and in vivo, thus highlighting a new role for this mechanism in the regulation of astrogliosis. The finding that IL-4 and IL-10 did not affect the NO/cGMP/calcium cascade or the GFAP response strongly suggests that this signalling is selectively involved in pro-inflammatory cytokine responses. This may help to gain a better understanding of the mechanism underlying the regulation of GFAP expression and may provide a possible target for controlling both physiological and pathophysiological processes in the CNS.

## Data Availability

All data are fully available.
